# Routine Transposition or In Situ Decompression? Rethinking Ulnar Nerve Strategy in Distal Humerus Fractures

**DOI:** 10.3390/jcm14207233

**Published:** 2025-10-14

**Authors:** Tahir Öztürk, Mete Gedikbaş, Fırat Erpala, Murat Aşçi

**Affiliations:** 1 Department of Orthopaedics and Traumatology, Memorial Bahçelievler Hospital, 34180 İstanbul, Turkey; ozturk.tahir@yahoo.com; 2 Department of Orthopaedics and Traumatology, School of Medicine, Bilecik Seyh Edebali University, 11230 Bilecik, Turkey; drmgedikbas@gmail.com; 3 Department of Orthopaedics and Traumatology, Çeşme Alper Çizgenakat State Hospital, 35930 İzmir, Turkey; drfiraterpala@hotmail.com

**Keywords:** distal humerus fracture, neuropathy, ulnar nerve, transposition

## Abstract

**Background**: Fractures of the distal humerus (DHFs) represent challenging intra-articular injuries that are often followed by postoperative complications, most notably ulnar neuropathy. There is still debate regarding the best method of handling the ulnar nerve during open reduction and internal fixation (ORIF). **Objective**: The primary objective was to evaluate the incidence of postoperative ulnar neuropathy (UN) in patients who underwent open reduction and internal fixation (ORIF) for DHFs, comparing anterior transposition (AT) with in situ decompression (ISD) of the ulnar nerve. Additionally, we investigated the influence of AT on individuals presenting with preoperative UN. **Methods**: A retrospective review was conducted on 68 patients (26 females and 42 males; mean age: 46.3 years) who underwent ORIF for intra-articular DHF between 2018 and 2022. Patients were divided into two groups: anterior transposition (*n* = 14) and in situ decompression (*n* = 54). Ulnar neuropathy was evaluated using the modified McGowan classification, and radiographic outcomes were assessed with AO/OTA fracture classification. **Results**: Sixty-eight patients (26F/42M) were included. The mean age was 46.3 years (20–77 years) and the mean follow-up time was 53 months (36–76 months). The postoperative UN incidence was 30.8% (21/68). Neuropathy was significantly higher in the transposition group compared to in situ decompression (57.1% vs. 24%; *p* = 0.012). Olecranon osteotomy (36.3% vs. 20%; *p* = 0.042) and parallel plate configuration (33.3% vs. 12.5%; *p* = 0.037) were also associated with increased neuropathy risk. Among patients with preoperative ulnar neuropathy (*n* = 12), functional recovery was more favorable with transposition, where 71% experienced full resolution compared to 60% in the in situ group. **Conclusions**: Routine anterior transposition of the ulnar nerve during ORIF for DHF is associated with an increased risk of postoperative neuropathy and should be avoided in patients without preoperative symptoms. However, transposition appears beneficial in patients with pre-existing ulnar neuropathy. Surgeons should individualize ulnar nerve management to balance surgical risks and neurological outcomes.

## 1. Introduction

Fractures of the distal humerus (DHF) account for 33% of elbow region fractures and approximately 5% of entire fractures [1,2,3]. Although rare, the majority (96%) are complex comminuted intra-articular fractures that are prone to various complications during and after surgery [4,5,6,7,8]. There is a bimodal age and gender distribution; in young male patients, it occurs after high-energy trauma, and in elderly female patients, it occurs after low-energy injuries, often resulting from falls while walking [3,4,9].

Currently, the gold standard treatment method for distal humerus fractures is open reduction and internal fixation (ORIF) with locking plates [8,10]. Although several surgical approaches have been proposed, the paratricipital approach for extra-articular fractures and olecranon osteotomy for intra-articular fractures are the most commonly used methods [11,12,13,14,15].

The leading complications after treatment of distal humerus fractures are ulnar neuropathy (UN) [16,17], implant irritation [18], and stiff elbow [19]. The fixed anatomic location of the ulnar nerve behind the medial epicondyle increases the potential for injury, both at the time of trauma and during surgical procedures and manipulations [17,20]. Reported rates of ulnar neuropathy following distal humerus fractures range around 12% on average, but rates ranging from 0% to 50% have been reported in the literature [21,22,23,24,25,26]. Although the ulnar nerve is frequently isolated during surgical procedures, there is still no consensus regarding the optimal management strategy during open reduction and internal fixation of distal humerus fractures in patients without preoperative neurological deficits [17,27].

We aimed to investigate the postoperative frequency of UN in patients treated with either anterior transposition or in situ decompression (ISD) of the ulnar nerve during ORIF of DHFs. The second purpose is to investigate the effect of anterior transposition (AT) in patients with presence of UN preoperatively.

## 2. Materials and Methods

### 2.1. Patient Selection

Ethical approval was obtained from the Ethics Committee of Tokat Gaziosmapaşa University School of Medicine (Decision No: 23-KAEK-009, Date: 19 January 2023). All patients provided written informed consent prior to their participation. The study was conducted in accordance with the principles of the Declaration of Helsinki.

Patients who underwent ORIF for intra-articular distal humerus fractures between January 2018 and January 2022 were included in the study. Eligible patients were older than 18 years and younger than 75 years.

Patients who had previously undergone surgery for the same elbow fracture or ulnar nerve transposition, those diagnosed with a stiff elbow, patients who had preoperative symptoms of UN or were diagnosed with cubital tunnel syndrome, and those with a follow-up period of less than three years were excluded from the study. In addition, AO Type B fractures were excluded because, by classification, they involve relatively small fragments affecting only a single column or the trochlea, are not routinely treated with standardized parallel plating, and could therefore potentially confound the results. The patients’ preoperative data, including the mechanism of injury, fracture type, and the presence of ulnar neuropathy, were obtained from their medical records.

Patients who met the inclusion criteria were divided into two groups: Group I comprised patients who underwent ulnar nerve transposition, and Group II comprised patients who underwent in situ decompression.

### 2.2. Functional and Radiologic Evaluations

The presence and severity of ulnar neuropathy were evaluated at the initial assessment following trauma and at the final follow-up examinations using the McGowan staging system [28]. Assessments were performed by an independent orthopedic surgeon. According to this classification, in Grade I lesions, there is no disturbance of motor functions, but paresthesia and dullness are observed in the area of sensory distribution of the ulnar nerve. Grade II lesions show weakness of the interosseous muscles and sensory limitation with moderate atrophy, whereas Grade III lesions show interosseous paralysis and marked hypoesthesia.

The AO/OTA classification system was used for classification of the fractures. Patients with AO/OTA Type A and Type C fractures were included in the study. At the final follow-up, anteroposterior and lateral radiographs were obtained to evaluate fracture consolidation and to detect possible complications, such as arthrosis, implant failure, and heterotopic ossification.

### 2.3. Surgical Technique

**Figure 1:**
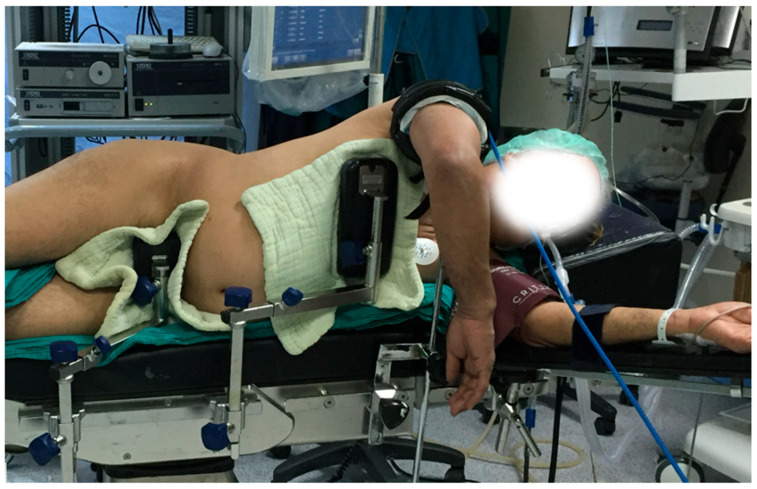
Positioning of the patient: after the patient has been placed in the lateral decubitus position, a tourniquet is applied as proximally as possible and an arm holder is placed under the elbow.

All patients underwent surgery in a single center by 2 different surgeons with 10 years (M.A.) and 5 years (T.Ö.) of experience in elbow surgery. Patients were placed in the lateral decubitus position. After a tourniquet was applied as proximally as possible to the extremity to be operated on, it was fixed with an arm holder (Figure 1). The operation began with a posterior midline incision. The ulnar nerve was carefully isolated and protected in the cubital tunnel. The fracture line was then accessed via a paratricipital approach or an olecranon osteotomy, depending on the type of fracture and the surgeon’s preference. Osteosynthesis was performed on all patients using anatomic elbow plates (Acumed, Hillsboro, OR, USA). After completion of the operation, osteosynthesis of olecranon osteotomy (if performed) was performed with tension band technique (cannulated screw (Synthes, DePuy, MA, USA) or Kirschner wires). The decision to perform either ISD or AT was made according to the nerve status and the surgeon’s choice intraoperatively. Intraoperatively, the surgeon assessed the position and stability of the ulnar nerve in relation to the plate within the cubital tunnel. If contact with the hardware or a risk of instability was identified, AT was carried out. In cases where the nerve could be repositioned securely without tension or impingement, ISD was preferred.

During anterior transposition, the nerve was completely released and identified distally between the two slips of the flexor carpi ulnaris. It was meticulously followed throughout the procedure and encircled with vessel loops for orientation. To ensure stability and prevent displacement, the nerve was placed beneath the fascial layer in the subcutaneous plane.

### 2.4. Statistical Analysis

Statistical analyses were performed using SPSS software (IBM Corp., Chicago, IL, USA, version 23.0). The normality of data distribution was tested with the Shapiro–Wilk test. Categorical variables were compared using Pearson’s chi-square, Fisher’s exact, or the Fisher–Freeman–Halton test, where appropriate. Continuous variables with normal distribution were analyzed using Student’s *t*-test, while non-normally distributed data were examined with the Mann–Whitney U test. For paired data that were not normally distributed, the Wilcoxon signed-rank test was applied. A *p*-value of less than 0.05 was accepted as the threshold for statistical significance.

## 3. Results

**Figure 2:**
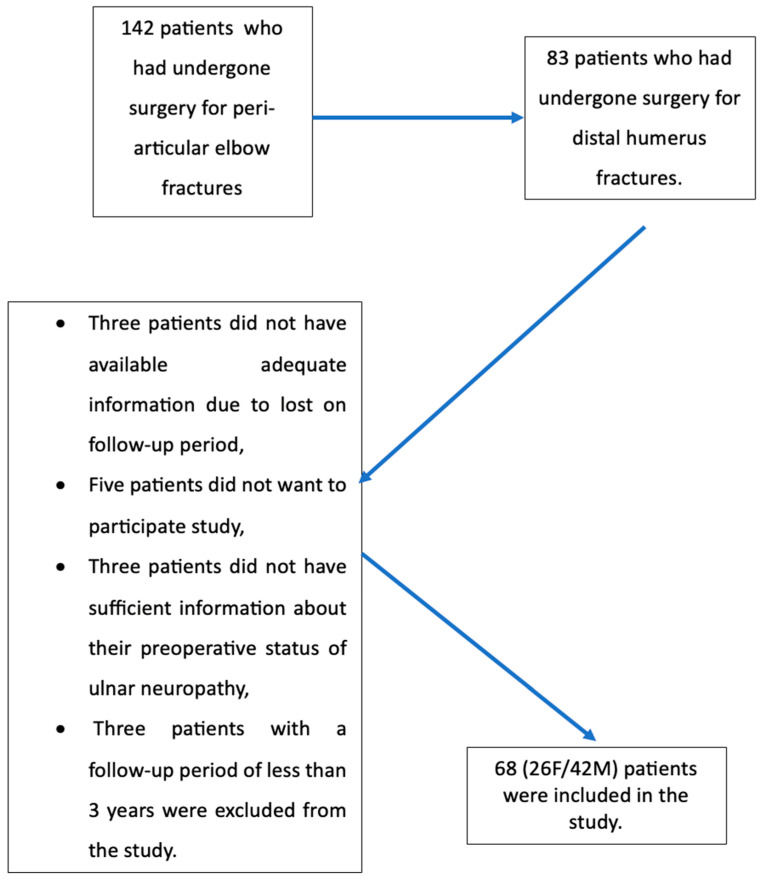
Presentation of the participating patients’ inclusion and exclusion. F: Female, M: Male.

Sixty-eight patients (26 female and 42 male) who met the inclusion criteria were included in the study. (Figure 2) The mean age was 46.3 years (20–77 years). The mean follow-up was 53 months (36–76 months). The mean time from trauma to surgery was 1.63 (0–4 days) (Table 1).

A total of 23 AO Type A, and 45 AO Type C fractures were identified. Olecranon osteotomy required 82.3% of the cohort. Of these, 42 (76%) were fixed with cannulated screw and 13 (24%) with Kirschner wires and a tension band. In eight patients, the plates were placed in a perpendicular configuration, while in 60, they were placed in a parallel configuration.

ISD was performed in 54 patients (79.5%) and AT was performed in 14 patients (20.5%). Ulnar neuropathy was detected in 30.8% of patients, with a significantly higher frequency in the AT cohort (57.1%, 8/13) compared to those treated with ISD (24%, 13/54) (*p* = 0.012). Of the 21 patients who developed UN, 13 (61.9%) had Grade I and 8 (38.1%) had Grade II UN. UN incidence was significantly higher 36.3% (16/44) in patients who underwent olecranon osteotomy and 20% (5/24) in patients who underwent surgery without olecranon osteotomy (*p* = 0.042). The frequency of UN is significantly higher in the parallel plate configuration. (12.5% vs. 33.3%; *p* = 0.037) (Table 2). Persistent ulnar neuropathy after anterior transposition led to reoperation in three patients, all classified as McGowan Grade II.

Twelve of the patients included in this study had preoperative symptoms of ulnar neuropathy. Of these, seven underwent anterior transposition and five underwent in situ decompression. At final follow-up, five of the patients who had undergone transposition had fully recovered, while two patients with Grade II neuropathy had improved to Grade I. Three of the five patients who had undergone in situ decompression made a full recovery, while two continued to experience symptoms of Grade I ulnar neuropathy (Table 3). Preoperative ulnar neuropathy symptoms demonstrated greater recovery in patients who underwent ulnar transposition (*p* = 0.042).

According to the AO/OTA classification, neuropathy was observed in five patients with type A fractures (all A3), and seven patients with type C fractures (three C1, two C2, and two C3). Although it was more frequent in type C fractures, no statistically significant difference was observed (*p* = 0.276). Similarly, at the final follow-up, although ulnar neuropathy was more frequently observed in AO type C fractures, the difference in incidence was not statistically significant (*p* = 0.999) (Table 4).

The overall complication rate was 48.5% (33/68). The most common adverse outcome was ulnar neuropathy of varying severity, identified in 21 patients (30.8%). Additional complications that may have affected the outcomes included four instances of nonunion (pseudoarthrosis) and three cases of postoperative wound infection. Furthermore, five patients developed elbow stiffness. In terms of reinterventions, five patients showed intolerance to the plate, screws, or K-wire, necessitating removal; two patients who developed nonunion after olecranon osteotomy were treated with grafting using autogenous tricortical and cancellous bone harvested from the iliac crest, and re-osteosynthesis was performed with plate fixation; one patient developed a superficial infection and required irrigation and debridement, after which the infection resolved without further complications; and six patients required ulnar nerve release due to persistent neuropathy that showed no clinical improvement.

## 4. Discussion

The results of this study show that ulnar neuropathy can develop in about one-third of patients after treatment of distal humeral fractures, depending on the type of fracture, olecranon osteotomy, transposition of the ulnar nerve, and the plate configuration. Treatment of distal humerus fractures is demanding, primarily due to the multifaceted fracture patterns and the close relationship to surrounding neurovascular structures [27]. The incidence of ulnar neuropathy in patients who have undergone surgery for DHF via the ORIF method ranges from 0% to 51% [20,29,30,31,32,33,34,35,36]. Ulnar neuropathy may occur due to several factors; iatrogenic factors include aggressive traction and manipulations for fracture realignment, implant irritation, and nerve devascularization during nerve dissection [37].

Many surgeons preserve the ulnar nerve after dissecting it at the beginning of surgery for distal humerus fractures. However, there is still no consensus on anterior transposition of the nerve [29]. Some authors recommend routine anterior transposition [15,32,38] while others recommend making the decision dependent on the preoperative status and whether the plate and nerve are in contact [23,39,40]. However, some publications state that transposition is not necessary [21,34,41]. In this study, anterior transposition was performed if deemed necessary, depending on the condition of the nerve during surgery.

Potential benefits of anterior transposition include that there is no contact with the plate, subluxation of the medial condyle is prevented, and the nerve is not compressed in the scar tissue that may develop [27,42]. Nevertheless, the likelihood of ulnar neuropathy may rise with the development of neuropraxia due to manipulation during transposition, injury to the feeding arteries during dissection, inadequate release, lack of adequate soft tissue support in the transfer area, and the development of fibrosis [27,43]. In contrast, in situ decompression is a simpler method, requiring less manipulation and carrying a lower risk of devascularization [27]. These predisposing factors explain the increased development of ulnar neuropathy in the transposition group in this study.

Although transposition has been proposed to provide a theoretical benefit, several studies have demonstrated that it does not lower the risk of postoperative UN. Wiggers et al. [26] evaluated 107 patients with distal humerus fractures and reported a 17% incidence of UN, regardless of whether AT was performed. Consistently, other reports have shown similar results between AT and ISD in preventing UN [17,42]. In contrast to these findings, Chen et al. [29]. reported a four-times increased risk for UN development in AT compared to ISD. Similarly, Ahmed et al. [44]. found that AT resulted in approximately five times higher risk of postoperative dysfunction.

Ulnar nerve management strategies after DHF in patients with preoperative neuropathy have become increasingly important following the study of Ruan et al. [2], who investigated the efficacy of ISD versus AT for the treatment of patients with preoperative UN symptoms and found that 80% of patients’ symptoms recovered completely in the AT group, despite this 57% of the patients in the ISD group recovered completely. These findings suggest that routine transposition may not be justified, and the decision should instead be guided by intraoperative considerations and individual patient factors. Nauth et al. [13] found sufficient evidence (Grade B) to favor AT in distal humeral fracture treatment in patients with pre-existing UN. Similarly, McKee et al. [22] studied 21 patients with preoperative symptoms of UN who underwent anterior transposition and reported complete or partial relief of symptoms in 17 patients. Twelve of the patients included in this study had preoperative symptoms of UN. Five patients who underwent transposition recovered completely, while two patients with Grade II neuropathy regressed to Grade I. Three of the five patients who underwent in situ decompression recovered completely, while two continued to exhibit Grade I ulnar neuropathy. Given these results, which are consistent with the literature, we suggest that anterior transposition is beneficial in patients with early signs of ulnar neuropathy, but transposition should be avoided in patients without these symptoms.

Previous studies have reported conflicting findings regarding the influence of fracture type on the development of ulnar neuropathy. Wiggers et al. [26] suggested that high-energy trauma and more complex osteosynthesis in AO/OTA Type C (bicolumnar) fractures may predispose patients to neuropathy compared with Type A and B fractures involving the capitellum and trochlea. In contrast, Helfet, Schmeling, and Worden et al. found no such association [17,45]. Consistent with the latter, despite the relatively high proportion of Type C fractures in our cohort, fracture type was not identified as a statistically significant risk factor for the development of neuropathy.

The limitations of this study are as follows: (1) it is a retrospective study, (2) the operations were performed by two different surgeons, (3) the number of patients was relatively small compared to previous studies, (4) the presence of UN was assessed using the McGowan classification system rather than objective methods such as electromyography or the Semmes–Weinstein monofilament test, and (5) the changes in elbow range of motion and the effects of elbow stiffness on ulnar nerve functions were not examined. However, this study presents several advantages: (1) the same implant brand was used in all patients by a single surgeon, eliminating the risk of bias that could arise from surgeons with different experience and implant characteristics; (2) the follow-up period was long; and (3) functional outcomes were evaluated by two independent evaluators.

## 5. Conclusions

Based on these findings, routine anterior transposition of the ulnar nerve during ORIF for distal humerus fractures is not recommended in patients without preoperative neuropathy, as it increases the risk of postoperative dysfunction. Surgeons should instead favor in situ decompression in asymptomatic patients and reserve anterior transposition for those with pre-existing neuropathy or when intraoperative findings indicate direct contact with or tension on the nerve.

## Figures and Tables

**Table 1:** Descriptive characteristics.

		*n*	%
Gender	Female	26	38.3
	Male	42	61.7
Injured extremity	Right	39	57.3
	Left	29	42.7
AO Classification Type	A (A2/A3)	23 (14/9)	33.9
	C (C1/C2/C3)	45 (7/23/15)	66.1
Ulnar nerve transposition	Yes	14	20.5
	No	54	79.5
Plate Configuration	90°	8	11.7
	180°	60	88.3
Olecranon Osteotomy	Yes	56	82.3
	No	12	17.7
Non-union	Yes	5	14.7
	No	29	85.3
Infection	Yes	4	11.7
	No	30	88.3
Preoperative Ulnar Neuropathy	0	56	82.3
	1	8	11.7
	2	4	6
Postoperative Ulnar Neuropathy Grade	0	47	64.7
	1	13	20.6
	2	8	14.7

**Table 2:** Investigation of the factors that influence the presence of ulnar neuropathy.

	Ulnar Neuropathy		
	Presence (*n* = 21)	Absence (*n* = 47)	*p*
Management strategy			0.012
Anterior transposition	8	2	
In situ decompression	13	2	
Plate configuration			0.037
180°	20	40	
90°	1	7	
Olecranon osteotomy			0.042
Not performed	5	19	
Performed	16	28	

**Table 3:** Evaluation of the effects of ulnar nerve treatment on the symptoms of patients with preoperative symptoms of ulnar neuropathy.

		Presence of Ulnar Neuropathy		
		Preoperative	Postoperative	*p*
Anterior transposition	Grade 1	4	2	0.042 **
	Grade 2	3	0	
In situ decompression	Grade 1	3	2	
	Grade 2	2	0	

Chi-square test, Fisher’s Exact test and ** Fisher–Freeman–Halton tests were performed.

**Table 4:** Relationship between the presence of ulnar neuropathy and the type of fracture.

		Fracture Type		
		Type A (*n*:23)	Type C (*n*:45)	*p*
Preoperative Ulnar Neuropathy	Yes	5	7	0.276 **
	No	18	38	
Postoperative Ulnar Neuropathy	Yes	7	14	0.999 **
	No	16	31	

** Fisher–Freeman–Halton test was performed.

## Data Availability

Patient data are available from the corresponding author when requested with a reasonable justification.

## Abbreviations

The following abbreviations are used in this manuscript:

DHF	Distal humerus fracture.
ORIF	Open reduction internal fixation.
AO/OTA	Orthopaedic Trauma Association.

## References

[1] Bogataj M., Kosel F., Norris R., Krkovic M., Brojan M. (2015). Biomechanical Study of Different Plate Configurations for Distal Humerus Osteosynthesis. Med. Biol. Eng. Comput..

[2] Ruan H.-J., Liu J.-J., Fan C.-Y., Jiang J., Zeng B.-F. (2009). Incidence, Management, and Prognosis of Early Ulnar Nerve Dysfunction in Type C Fractures of Distal Humerus. J. Trauma Acute Care Surg..

[3] Dey Hazra R.-O., Lill H., Jensen G., Imrecke J., Ellwein A. (2018). Fracture-Pattern-Related Therapy Concepts in Distal Humeral Fractures. Obere Extrem..

[4] Beazley J.C., Baraza N., Jordan R., Modi C.S. (2017). Distal Humeral Fractures-Current Concepts. Open Orthop. J..

[5] Savvidou O.D., Zampeli F., Koutsouradis P., Chloros G.D., Kaspiris A., Sourmelis S., Papagelopoulos P.J. (2018). Complications of Open Reduction and Internal Fixation of Distal Humerus Fractures. EFORT Open Rev..

[6] McKee M.D., Veillette C.J., Hall J.A., Schemitsch E.H., Wild L.M., McCormack R., Perey B., Goetz T., Zomar M., Moon K. (2009). A Multicenter, Prospective, Randomized, Controlled Trial of Open Reduction—Internal Fixation versus Total Elbow Arthroplasty for Displaced Intra-Articular Distal Humeral Fractures in Elderly Patients. J. Shoulder Elb. Surg..

[7] Lawrence T.M., Ahmadi S., Morrey B.F., Sánchez-Sotelo J. (2014). Wound Complications after Distal Humerus Fracture Fixation: Incidence, Risk Factors, and Outcome. J. Shoulder Elb. Surg..

[8] Patel J., Motwani G., Shah H., Daveshwar R. (2017). Outcome after Internal Fixation of Intraarticular Distal Humerus (AO Type B & C) Fractures: Preliminary Results with Anatomical Distal Humerus LCP System. J. Clin. Orthop. Trauma.

[9] Ogasawara M., Tanaka H., Tsukano H., Tashima H., Yamamoto T. (2025). Incidence and Risk Factors of Ulnar Neuropathy After the Surgical Treatment of Distal Humeral Fractures. Cureus.

[10] Lauder A., Richard M.J. (2020). Management of Distal Humerus Fractures. Eur. J. Orthop. Surg. Traumatol..

[11] Patiño J., Rullan Corna A., Abdon I., Michelini A., Mora Pulido D. (2021). Paratricipital Approach for Distal Humerus Fractures. Musculoskelet. Surg..

[12] Saracco M., Smimmo A., De Marco D., Palmacci O., Malerba G., Vitiello R., Maccauro G., Minutillo F., Rovere G. (2020). Surgical Approach for Fracture of Distal Humerus: Posterior vs Lateral. Orthop. Rev..

[13] Nauth A., McKee M.D., Ristevski B., Hall J., Schemitsch E.H. (2011). Distal Humeral Fractures in Adults. J. Bone Jt. Surg..

[14] Jeong H.-S., Yang J.Y., Jeon S.J., Shon H.-C., Oh J.-K., Lim E.J. (2022). Comparison of Olecranon Osteotomy and Paratricipital Approach in Distal Humerus Intra-Articular Fracture: A Systematic Review and Meta-Analysis. Medicine.

[15] Kinik H., Atalar H., Mergen E. (1999). Management of Distal Humerus Fractures in Adults. Arch. Orthop. Trauma Surg..

[16] Rosenlund A.-M.N., Søreide E., Madsen J.E., Flugsrud G.B., Douglass B.W., Midtgaard K.S. (2022). Outcomes and Complications after Open Reduction and Internal Fixation of Distal Humeral Fractures with Precontoured Locking Plates. OTA Int..

[17] Worden A., Ilyas A.M. (2012). Ulnar Neuropathy Following Distal Humerus Fracture Fixation. Orthop. Clin..

[18] Duckworth A.D., Clement N.D., White T.O., McQueen M.M. (2017). Plate versus Tension-Band Wire Fixation for Olecranon Fractures: A Prospective Randomized Trial. J. Bone Jt. Surg..

[19] Mihas A.K., Reed L.A., Patch D.A., Cimino A., Davis W.T., Young M., Spitler C.A. (2024). Risk Factors for Dysfunctional Elbow Stiffness Following Operative Fixation of Distal Humerus Fractures. J. Shoulder Elb. Surg..

[20] Nyman E., Dahlin L.B. (2024). The Unpredictable Ulnar Nerve—Ulnar Nerve Entrapment from Anatomical, Pathophysiological, and Biopsychosocial Aspects. Diagnostics.

[21] Doornberg J.N., Van Duijn P.J., Linzel D., Ring D.C., Zurakowski D., Marti R.K., Kloen P. (2007). Surgical Treatment of Intra-Articular Fractures of the Distal Part of the Humerus: Functional Outcome after Twelve to Thirty Years. J. Bone Jt. Surg..

[22] MCKee M.D., Jupiter J.B., Bosse G., Goodman L. (1998). Outcome of Ulnar Neurolysis during Post-Traumatic ction of the Elbow. J. Bone Jt. Surg. Br. Vol..

[23] Kundel K., Braun W., Wieberneit J., Rüter A. (1996). Intraarticular Distal Humerus Fractures: Factors Affecting Functional Outcome. Clin. Orthop. Relat. Res..

[24] Holdsworth B.J., Mossad M. (1990). Fractures of the Adult Distal Humerus. Elbow Function after Internal Fixation. J. Bone Jt. Surg. Br. Vol..

[25] Robinson C.M., Hill R.M., Jacobs N., Dall G. (2003). Adult Distal Humeral Metaphyseal Fractures: Epidemiology and Results of Treatment. J. Orthop. Trauma.

[26] Wiggers J.K., Brouwer K.M., Helmerhorst G.T., Ring D. (2012). Predictors of Diagnosis of Ulnar Neuropathy after Surgically Treated Distal Humerus Fractures. J. Hand Surg..

[27] García-Cepeda I., Sanz-Peñas A.-E., de Blas-Sanz I., Simón-Pérez C., Frutos-Reoyo E.-J., Aguado-Maestro I. (2025). Post-Surgical Ulnar Nerve Neuropathy in Distal Humerus Fractures: Comparison Between in Situ Decompression and Anterior Subcutaneous Transposition. J. Clin. Med..

[28] Mcgowan A.J. (1950). The Results of Transposition of the Ulnar Nerve for Traumatic Ulnar Neuritis. J. Bone Jt. Surg. Br. Vol..

[29] Chen R.C., Harris D.J., Leduc S., Borrelli J.J., Tornetta P., Ricci W.M. (2010). Is Ulnar Nerve Transposition Beneficial during Open Reduction Internal Fixation of Distal Humerus Fractures?. J. Orthop. Trauma.

[30] Yamaguchi K., Sweet F.A., Bindra R., Gelberman R.H. (1999). The Extraneural and Intraneural Arterial Anatomy of the Ulnar Nerve at the Elbow. J. Shoulder Elb. Surg..

[31] Henley M.B., Bone L.B., Parker B. (1987). Operative Management of Intra-Articular Fractures of the Distal Humerus. J. Orthop. Trauma.

[32] Gofton W.T., MacDermid J.C., Patterson S.D., Faber K.J., King G.J. (2003). Functional Outcome of AO Type C Distal Humeral Fractures. J. Hand Surg..

[33] Ek E.T., Goldwasser M., Bonomo A.L. (2008). Functional Outcome of Complex Intercondylar Fractures of the Distal Humerus Treated through a Triceps-Sparing Approach. J. Shoulder Elb. Surg..

[34] Aslam N., Willett K. (2004). Functional Outcome Following Internal Fixation of Intraarticular Fractures of the Distal Humerus (AO Type C). Acta Orthop. Belg..

[35] McKee M.D., Wilson T.L., Winston L., Schemitsch E.H., Richards R.R. (2000). Functional Outcome Following Surgical Treatment of Intra-Articular Distal Humeral Fractures through a Posterior Approach. J. Bone Jt. Surg..

[36] Sanchez-Sotelo J., Torchia M.E., O’Driscoll S.W. (2007). Complex Distal Humeral Fractures: Internal Fixation with a Principle-Based Parallel-Plate Technique. J. Bone Jt. Surg..

[37] Shearin J.W., Chapman T.R., Miller A., Ilyas A.M. (2018). Ulnar Nerve Management with Distal Humerus Fracture Fixation: A Meta-Analysis. Hand Clin..

[38] Athwal G.S., Hoxie S.C., Rispoli D.M., Steinmann S.P. (2009). Precontoured Parallel Plate Fixation of AO/OTA Type C Distal Humerus Fractures. J. Orthop. Trauma.

[39] Russell G.V., Jarrett C.A., Jones C.B., Cole P.A., Gates J. (2005). Management of Distal Humerus Fractures with Minifragment Fixation. J. Orthop. Trauma.

[40] Greiner S., Haas N., Bail H. (2008). Outcome after Open Reduction and Angular Stable Internal Fixation for Supra-Intercondylar Fractures of the Distal Humerus: Preliminary Results with the LCP Distal Humerus System. Arch. Orthop. Trauma Surg..

[41] Srinivasan K., Agarwal M., Matthews S.J., Giannoudis P. (2005). Fractures of the Distal Humerus in the Elderly: Is Internal Fixation the Treatment of Choice?. Clin. Orthop. Relat. Res..

[42] Vazquez O., Rutgers M., Ring D.C., Walsh M., Egol K.A. (2010). Fate of the Ulnar Nerve after Operative Fixation of Distal Humerus Fractures. J. Orthop. Trauma.

[43] Krkovič M., Kordaš M., Tonin M., Bošnjak R. (2006). Subperiosteal Elevation of the Ulnar Nerve during Internal Fixation for Fractures of the Distal Humerus Assessed by Intra-Operative Neurophysiological Monitoring. J. Bone Jt. Surg. Br. Vol..

[44] Ahmed A.F., Parambathkandi A.M., Kong W.J.G., Salameh M., Mudawi A., Abousamhadaneh M., Abuodeh Y., Ahmed G.O. (2020). The Role of Ulnar Nerve Subcutaneous Anterior Transposition during Open Reduction and Internal Fixation of Distal Humerus Fractures: A Retrospective Cohort Study. Int. Orthop..

[45] Helfet D.L., Schmeling G.J. (1993). Bicondylar Intraarticular Fractures of the Distal Humerus in Adults. Clin. Orthop. Relat. Res..

